# ‘PEN’ appendicitis

**DOI:** 10.4103/0971-9261.70649

**Published:** 2010

**Authors:** Gabriel Ngom, Issa Amadou, Olivier Ngaringuem, Oumar Ndour

**Affiliations:** Department of Pediatric Surgery, Aristide Le Dantec Hospital, Dakar, Senegal

**Keywords:** Appendicitis, foreign body, pen

## Abstract

We report a three-year-old boy who ingested the tip of a pen and presented with signs of appendicitis. Plain abdominal radiographs showed the foreign body in the right iliac fossa. Surgical exploration revealed perforated appendix and the foreign body in its lumen. Appendectomy resulted in satisfactory recovery.

## INTRODUCTION

Appendicitis due to a foreign body is a well-known entity. However it is particularly rare, estimated at about 0.0005% of all appendicitis.[1] It is most often caused by foam or sharp objects like toothpicks, pins, stones, bullets needles, etc.[1–3] We report a case of appendicitis in a boy aged three years caused by the tip of a pen. To our knowledge this is the first report of a foreign-body appendicitis caused by a pen.

## CASE REPORT

**Figure 1:**
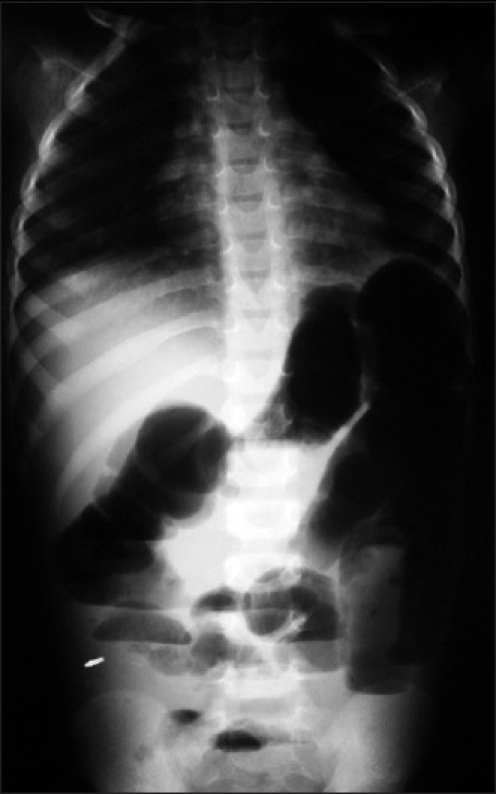
Tip of a pen in the right iliac fossa

A three-year-old boy was admitted for a persistent abdominal pain for four days without vomiting and bowel transit disorders. On examination, the child was afebrile. The abdomen was distended with tenderness and guarding in right iliac fossa. The laboratory tests were normal. Plain radiograph of the abdomen showed air-fluid level and a foreign body in the right iliac fossa, which seems to be a tip of a pen [Figure 1]. A second radiograph of the abdomen revealed the same image of foreign body always in right iliac fossa. But its localization was unknown. Given the strong suspicion of appendicitis, surgical exploration through Mc Burney incision was decided. We found appendicitis with perforation of the distal end of the appendix by the tip of the pen. The child recovered after appendectomy and is well six months later. The histological examination showed feature of appendicitis.

## DISCUSSION

The majority of ingested foreign bodies pass through the digestive tract without complication.[4] The most commonly reported complications are perforation (1%) and rarely appendicitis.[1,4] Many foreign bodies are described as being the cause of appendicitis particularly sharp pointed objects.[5] There has been one report of appendicitis due to screw.[6] The preoperative diagnosis was that of acute appendicitis. But we were not sure that the foreign body was responsible. The history taken from the child and parents did not reveal foreign body ingestion. However, we could suggest this diagnosis with the presence of foreign body in the right iliac fossa at the same level on two successive images of abdominal radiographs. In the case of foreign body appendicitis, appendectomy should be performed quickly because of complications associated with delayed diagnosis,[1] as was the case in our child who presented with perforation.
